# Relics and Historical Uses of Human Zootherapeutic Products in Contemporary Spanish Ethnoveterinary Medicine

**DOI:** 10.3390/vetsci8120323

**Published:** 2021-12-11

**Authors:** José A. González, José Ramón Vallejo

**Affiliations:** 1 Grupo de Investigación de Recursos Etnobiológicos del Duero-Douro (GRIRED), Facultad de Biología, Universidad de Salamanca, E-37071 Salamanca, Spain; ja.gonzalez@usal.es; 2 Departamento de Anatomía Patológica, Biología Celular, Histología, Historia de la Ciencia, Medicina Legal y Forense y Toxicología, Área de Historia de la Ciencia, Facultad de Medicina, Universidad de Cádiz, E-11003 Cádiz, Spain

**Keywords:** human being, ethnozoology, zootherapy, veterinary practices, Spain

## Abstract

(1) Background: this review documents the wide repertoire of practices and remedies based on the use of human-derived products in Spanish ethnoveterinary medicine (EVM) from the early 20th century to the present. These practices are compared with historical data and those of other countries; (2) Methods: a search using advanced functions in the most important databases in the fields of ethnobiology, EVM, folklore, and ethnography was performed. Information was obtained from 29 documentary sources; (3) Results: from the search of the literature, 46 use-reports related to the veterinary use of human urine, menstrual fluid, saliva, breast milk, and faeces were recorded. These zootherapeutic resources are/were used to treat 20 animal diseases, in particular dermatological ailments. In addition, many practices of the magical–religious type are documented; (4) Conclusions: the veterinary uses described and analysed here are fundamental to the development of therapeutic tools and creating teaching and learning processes in new popular veterinary practices adapted to the users and those who demand them. The information collected could form a scientific foundation for future inventories of local veterinary knowledge (LVK) and research addressing the discovery of new drugs for livestock. This work contributes to the inventory of some uses, traditional practices, and rituals seriously threatened by the progressive loss of LVK in Europe.

## 1. Introduction

Ethnoveterinary medicine (EVM) is a scientific discipline that focuses on people’s knowledge of animal diseases and their control, practices, and remedies for the treatment and prevention of animal diseases; it also includes the management strategies and spiritual elements related to the care and production of animal health [1,2]. It is currently an important scientific field with an increasing number of research projects. As a result, the publication of studies on traditional practices aimed at curing and/or preventing diseases in domestic animals is increasingly more frequent, particularly in international journals on veterinary medicine or pharmacology [3,4]. Only taking into consideration studies published in the English language, notable examples can be found in Asia [5,6,7], Africa [8,9], Latin America [10], and Europe [11,12,13].

In the specific case of Spain, the development of the Spanish Inventory of Traditional Knowledge (IECT, in Spanish the *Inventario Español de los Conocimientos Tradicionales*) has enabled the documentation of the use of 711 vascular plant species as veterinary herbal medicine [14]. This large number of phytotherapeutic resources indicates the existence in our country of a very rich biocultural heritage related to EVM and to animal health care in general. However, Spanish EVM is not only based on the use of herbal remedies. As in other parts of the world [10,12,15], different animal body parts, secretions, and derivative products have traditionally been used for veterinary purposes. As such, in a trilogy of review articles, the traditional veterinary use of 18 species and five ethnotaxa of invertebrates [16], 30 wild vertebrate species [17], and nine domestic animal species, including one hybrid (the mule) [18], can be found.

During the research and data collection stage regarding different animal species for veterinary use, in some of the many documentary sources consulted (theses, books, journal papers), we found a collection of references on the veterinary use of different human-derived products. The analysis of these references led us into an epistemological debate on the systemisation of zootherapeutic resources. In this respect, it should be noted that many authors include our own species, *Homo sapiens*, in the lists of animal species used for veterinary (or medical) purposes in their publications [19,20]. However, we consider that the use of zootherapeutics of human origin presents a singularity and some historical–anthropological peculiarities that deserve to be published separately. Indeed, thanks to disciplines such as the history of medicine or the anthropology of health, we know of magical–religious remedies based on organs, secretions, or remains where the symbolism of the human species or its activities is decisive [21,22]. In this way, by categorising and segregating human zootherapeutic resources, we do not seek to establish an anthropocentric criterion, but wish to make an ethnographic approach consistent with the history of humanity and its traditions from an ethical and cross-cultural perspective [23,24]. Hence, our position is based on beliefs such as the healing power of human blood, which led to the use of the blood of dead gladiators as an anti-epileptic agent, a practice that continued for centuries with beheadings [21] and, more recently, the cruel murder of children to use their blood as an opioid medicine against consumption [25] (p. 287). On the other hand, looking specifically at our species as a therapeutic resource allows us to examine more deeply and reflect upon the magical thinking present in societies of any culture and time. There is no doubt that the human body is an excellent subject for study in order to delve into ancestral healing practices, the use of rituals, fetishes, amulets, or talismans, and to establish a dialogue between history, anthropology, and the health sciences [26,27,28,29,30]. Moreover, beliefs are so particular that the segregation of our species from the other animal species can assist us in reflecting on the “One medicine, one health” approach [31,32] through the comparison of traditional veterinary and medical practices and knowledge.

Thus, the aims of the present review are as follows: (1) to report and analyse local knowledge about the veterinary use of human beings and their derivative products (body fluids, excrements) in contemporary Spanish EVM; (2) to contribute to the transfer of part of the traditional ecological knowledge to new generations; (3) to contribute to the dissemination of results within the scientific community in order to open a door to research in other disciplines, such as epidemiological studies of the anthropology of health or livestock sociology; and (4) to contribute to the management and conservation of the biocultural heritage of Spain.

## 2. Materials and Methods

### 2.1. Data Collection

As already mentioned in the previous narrative review articles [16,17,18], to access the maximum number of documentary sources, a search using advanced functions in the most important international and national databases was conducted. However, being aware of the large amount of data on ethnoveterinary uses scattered in the secondary scientific literature, a survey was conducted to allow access to local information and relevant non-indexed literature. The procedure was implemented by recruiting and analysing pertinent secondary literature to make the inventory as complete as possible. The ISI Web of Science and Anthropology Plus and JSTOR III—Arts and Sciences international databases were consulted. The national resources referenced include the database of Ph.D. Theses, TESEO; the information system of the databases of the CSIC (Spanish Research Council); the Dialnet bibliographic website; Google Scholar; and the catalogue of Public State Libraries. The overall search pattern covered the title, abstract, and keywords concerning ethnozoology-related disciplines that have UNESCO codes (e.g., anthropology, the history of veterinary science, and zoology) and the terms human, human being, *Homo sapiens*, livestock, folk veterinary medicine, folklore, ethnobiology, ethnozoology, ethnoveterinary medicine, and zootherapy, in conjunction with the Spanish geographical context. No restrictions regarding the language of the publications consulted were imposed.

### 2.2. Presentation of the Data

In contrast to the previous review articles [16,17,18] in which the veterinary remedies documented were organised into main categories of therapeutic use based on the organ systems of the animal body, such as the digestive, respiratory, or reproductive system (infectious livestock diseases, parasite-induced ailments, or poisoning are also taken into consideration as use categories), in this review the documented remedies are arranged according to the product used.

Mention will be made in all cases of the human product used, the disease or trouble treated, the group(s) of domestic animals treated, the route of administration, the place or region of origin of the collected data, and the documentary source.

## 3. Results and Discussion

### 3.1. Documentary Sources

This review was carried out using data including 29 documentary sources from the beginning of the 20th century to the present day. Among them, 23 have allowed for the registration of 25 empirical remedies based on the use of different products of human origin. As for the type of these 23 sources, we have obtained use-reports from nine journal papers, all published in journals in the field of folklore and ethnography, and 13 books, five concerning superstition, folklore, or ethnobotany, four within the scope of ethnomedicine, and only another four directly related to EVM. We also obtained data from one Ph.D. thesis approaching the study of EVM in a particular geographical area.

In order to assess how contemporary the reviewed veterinary practices are, we mainly obtained use-reports in studies published over the past 20 years, namely from eight works published between 2000 and 2009 and four published between 2010 and 2020. We have also included data collected in five works from the 1990s, three from the 1980s, and three from the first half of the 20th century (years 1931, 1935, and 1949).

### 3.2. Quantitative Ethnozoological/Ethnoveterinary Analysis

A total of 46 use-reports related to the veterinary use of the four human bodily fluids (urine, menstrual fluid, saliva, and breast milk) and our excreta, human faeces, were recorded in Spanish EVM to treat a total of 20 animal diseases or medical conditions. Table 1 summarises the products used and the most relevant information of the veterinary remedies documented.

These therapeutic resources of human origin are usually applied in simple ways, mostly through the direct application to the affected area or by ingestion, and typically not in association with other animal-derived ingredients. In some cases, however, an association with medicinal plants or chemical products is observed. Remedies are applied externally in 19 cases (76%) and internally in six (24%).

### 3.3. Traditional Veterinary Medicinal Practices

**Figure 1:**
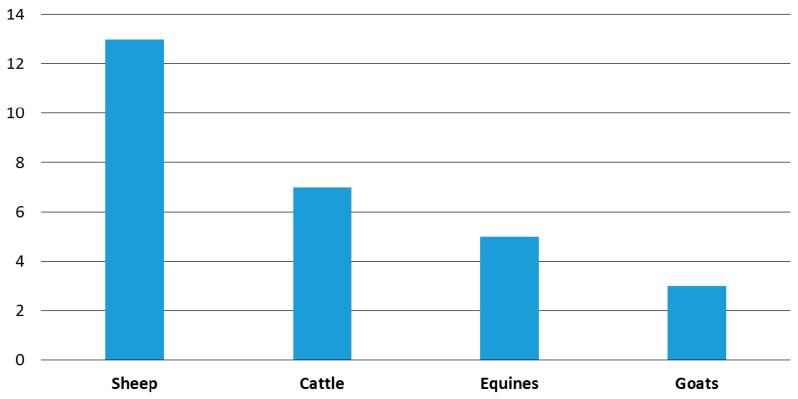
Number of empirical remedies documented for the treatment of specific domestic animal groups.

Only one of the 25 documented remedies is described by the authors consulted as “used to treat all livestock” in general and not for any single domestic animal group in particular. In accordance with their economic importance and as working animals (and with the sole exception of a remedy for curing cough in piglets), sheep, cattle, equines, and goats are the groups of domestic animals for which remedies were documented (Figure 1).

The most reports were regarding treatment in sheep. This is a truly interesting fact that reflects how the daily care of the herd, the in situ action of the shepherds in case of injury to their animals in the countryside or the characteristics of pastoralism in Spain (transhumance) [37,38,40,54] translate into a large number of ovine ethno-therapeutic resources.

**Figure 2:**
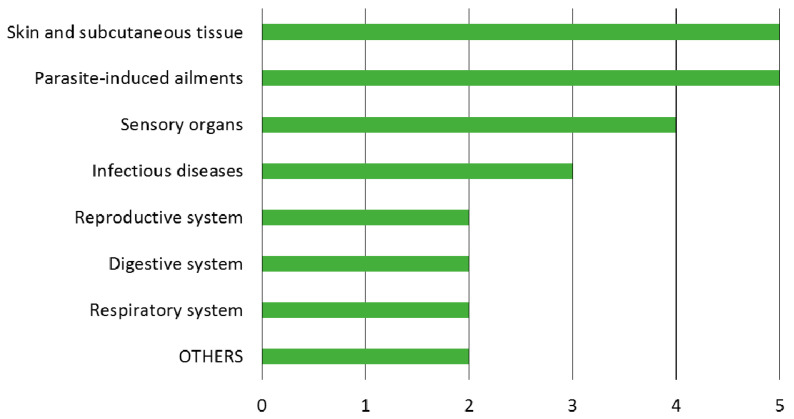
Number of empirical remedies documented used per disease category.

The remedies refer to seven main categories of therapeutic use (Figure 2). The most frequent indications are diseases of the skin and subcutaneous tissue (5 remedies), in particular for wound healing, parasite-induced ailments (5), and disorders of the sensory organs (4).

Below is a list of the documented remedies, arranged according to the product used.

#### 3.3.1. Urine

To treat anthrax in sheep and goats in Valsequillo (Gran Canary Island), human urine was administered to sick animals [33]. In the Basque Country, they treated cough in piglets by giving them a small amount of children’s urine to drink [34,35]. Similarly, in Galicia, they would give sick animals human urine to drink each morning for eight days to treat cases of cough in cows [36]. Shepherds in Extremadura [37], as well as those as far away as San Mateo, in the Canary Islands [33], treated mastitis in their ewes by urinating on the diseased udders and scrubbing them well with the urine; in addition, rubbing the udders of ewes with children’s urine was the remedy of choice for cleaning and healing wounds and cracks in Extremadura [37,38]. Regarding the field of veterinary entomology, to combat sheep body lice (*Bovicola ovis*) in Extremadura, urine was used as a topical application [37,38]; further, in the Valle de Carranza (Vizcaya), cutaneous myiasis in cows was treated by rubbing the animal with soap and urine. The urine used on this last case had to be warmed before use, and after the treatment, the animal was covered with a blanket. In this way, the blowfly larvae, known locally as *barros*, were asphyxiated and, after a few days, would fall off [39]. Another very similar remedy consisted of boiling the leaves and bark of black elder (*Sambucus nigra* L.) in human urine and then rubbing an animal with the liquid obtained. After this action, the animal was also covered with a blanket [39]. To treat mange, the shepherds in Anguiano (La Rioja) washed their sheep and goats thoroughly with human urine [40]. Galician cowherders applied cloths soaked in urine to the eyes of their cows affected with pinkeye (infectious bovine keratoconjunctivitis) [36,41]. In the northern part of the province of Cáceres, a method for healing different eye injuries (cuts, ulcers, etc.) in sheep was to apply, as eye drops, children’s urine [37,38]. The inhabitants of A Pastoriza (Lugo) impregnated any wound in their cows with urine [42]. In Aldearrubia (Salamanca), if a farmer made a wound with the tip of a ploughshare on the leg of a cow or ox while ploughing, he would urinate on the wound [43]. It was very common during long days working in the countryside that equines would get injured or chafed by their harnesses; to prevent any infection or other type of wound, people in the district of La Campiña (Guadalajara) would wash them with plenty of urine [44]. In the same way, to disinfect any injuries on their sheep, the shepherds in Extremadura [37,38], or those in Bardenas Reales, Navarra [45], did not hesitate to urinate on the wounds.

Indeed, the region of Extremadura is rich in use-reports relating to the veterinary use of human urine. As such, the shepherds did not shrink from urinating on the wounds of their sheep to disinfect them, using “rotten urine” on their skin ulcers [37], or applying children’s recent urine to burns on their sheep [37,38]. Human urine was also used in this region of western Spain as an oral antidote for all livestock in the case of poisoning from ingesting hemlock (*Conium maculatum* L.) [46].

#### 3.3.2. Menstrual Fluid

In Monzón (Huesca), the topical use, or direct application, of this human product was used to treat sores and injuries in equines caused by the chafing of farm implements, and the wounds known as *higos* (figs), which are the lumps that sometimes appear on the ears of equines [52]. In Galicia, they used to rub warts on cows with menstrual blood for several days [36].

However, menstrual fluid has been used in Spanish EVM particularly as a therapeutic resource against equine colic, known popularly in Spanish as *torzón* or *tarazón*. In Asturias, equines were treated with women’s menstrual fluid dissolved in water to drink [47]. In the Sierra de Cádiz [48] and in Malpartida de Plasencia (Cáceres) [49], a sick animal was given to drink, “with good results”, the water from washing the pants and cloths used by women as sanitary towels marked with the fluid from when the women were menstruating. Similarly, in Galicia, they would rub the painful area of an animal with the shirt worn by a woman while menstruating, afterwards washing the item and giving the equine patient the water from washing it to drink [36,41,50,51].

#### 3.3.3. Saliva

Shepherds in Treviño (Burgos) treated scald (hoof rot) in their sheep by throwing a little saliva over their hooves and rubbing it thoroughly all over them [53]. In Zarza de Granadilla (Cáceres), sheep scab (mange) was treated by applying a mixture of turpentine, sheep tallow, and human saliva [37,38]. Corneal ulcers in sheep and goats were treated, and in some cases still are treated, by applying human saliva into the damaged eye. This is a remedy documented in areas of Spain as far apart as Tierra de Cameros (La Rioja) [40], Campo de Montiel (Albacete) [54], and Cueva Corcho (Gran Canary Island) [33].

#### 3.3.4. Breast Milk (or Mothers’ Milk)

For many shepherds in Extremadura, the most effective treatment to relieve their sheep suffering from otalgia was to pour into the sick animal’s ear a few drops of human breast milk [37].

#### 3.3.5. Excrement (Human Faeces)

In Serradilla (Cáceres), the grazes, sores, and wounds caused by leather harnesses in working and draft equines were treated by applying a poultice of human excrement [49]. In cases of bloat, in many areas of Cantabria, cows were forced to swallow a handful of human excrement. The purpose of this scatological practice was to provoke vomiting, thereby relieving the animal [55].

## 4. Potential Veterinary and Pharmacological Uses

### 4.1. Urine

Human urine is composed primarily of water (95%). The rest is urea (2%), creatinine (0.1%), uric acid (0.03%), chloride, sodium, potassium, sulphate, ammonium, phosphate, and other ions and molecules in lesser amounts. Protein is only found in trace amounts compared to their values in blood plasma [56,57].

It is important to note that the ammonia excreted in urine promotes acid excretion, so it seems that the existence of this substance in urine instinctively led to its use as a pharmacological element, particularly in dermatology [58]. Recent work currently confirms the activity of human urine in wound healing in rats compared with an antiseptic agent. The study concludes that human urine has significant wound healing activity in the excision, incision, burn, and dead space wound model [59]. In the same way, research using urine stem-cell-based therapy is encouraged as an innovative strategy for accelerating wound closure and promoting angiogenesis. In fact, human urine-derived stem cells, together with polycaprolactone/gelatin, have now been proven to be effective in the healing of full-thickness skin wounds in rabbits [60].

### 4.2. Menstrual Fluid

Menstrual fluid contains blood, cells from the lining of the uterus (endometrial cells), and mucus. It is important to note that it is a unique body fluid that contains mesenchymal stem cells. This fact is of great relevance for its therapeutic applications, derived from its biological activity, in the clinic for certain diseases and as an excellent tool for regenerative medicine, as it has been used successfully in cartilage regeneration [61]. In addition, studies have shown evidence of this product improving skin regeneration processes in vivo and in wound healing [62,63], offering excellent prospects for skin repair in difficult-to-heal conditions [63]. On the other hand, it has also been shown that the interaction of menstrual-blood-derived stem cells (MenSCs) with an inflammatory environment could enhance their beneficial properties [62]. In summary, there are very good pharmacological prospects and potentialities in MenSCs, which are gradually being described in the scientific literature; Chen and colleagues [64] review the therapeutic effects of MenSCs on a good number of conditions, including liver disease, diabetes, stroke, Duchenne muscular dystrophy, ovarian-related disease, myocardial infarction, Asherman syndrome, Alzheimer’s disease, acute lung injury, cutaneous wounds, endometriosis, and neurodegenerative diseases.

### 4.3. Saliva

Human saliva mainly consists of water (99%) and it is rich in antimicrobial compounds such as hydrogen peroxide, lactoferrin, and lysozymes. However, the most relevant antimicrobial components are peptides, which form the first main line of defense [65]. Among these we can mention defensins, cathelicidins, and histatins in the important synergistic action that is a limiting factor for microbial growth [65,66]. The presence of the group of antimicrobial peptides known as cathelicidins is very important for its involvement in popular therapy: they are essential in wound healing, immunomodulation, and angiogenesis [65,67]. For the same reason, histatins are of great interest as they possess wound healing properties essential in the processes of re-epithelialisation and endothelial cell adhesion [65,68,69]. Likewise, opiorphin is present in saliva and has analgesic properties that block pain signals and prevent the degradation of substances that activate opioid receptors [65,70]. Saliva could clearly improve wound healing, which is related to its effects on reducing inflammatory cell infiltration, preventing the infection of wounds, accelerating collagen fibre proliferation, and promoting vessel reconstruction in the wound healing process. Moreover, for Rodrigues Neves and colleagues [71], saliva has a great therapeutic potential for the treatment of open skin wounds. In their study that was performed in vitro to investigate the proliferation and migration of keratinocytes from the skin and gums, they show that human saliva can stimulate skin wound closure and an inflammatory response.

Finally, according to Vila and colleagues [65], who relate the components and functions of saliva, it can be noted that wound healing would be linked to growth factors, histatins, secretory leukocyte protease inhibitor, trefoil factor, leptin, its antibacterial capacity with amylases, cystatins, histatins, mucins, peroxidases, and its antifungal properties with histatin 5, β-defensins, and cathelicidins.

### 4.4. Breast Milk

Breast milk has very special characteristics. Similar to an unstructured living tissue, like blood, it can transport nutrients, enhance immunity, destroy pathogens, and influence biochemical systems. Its composition includes a large number of bioactive products such as phagocytes, lymphocytes, lipase, lactoferrin, and lysozyme, the bactericidal and anti-inflammatory action of which is very relevant for infant development and the immune system [72,73]. In principle, its biological activity seems to be decisive in otitis as it has been used in therapeutic practices throughout history. However, its bactericidal properties could only be effective in the external part of the ear canal as breast milk cannot cross the eardrum barrier. This is a problem, as most ear infections are middle ear infections and would disappear on their own. The discovery of growth factors, cytokines, the presence of stem cells, and probiotic bacteria has stimulated interest in research into the use of human breast milk as a medicine. As such, in a recent review [74], its effect was described on the treatment of conjunctivitis, chapped nipples, rhinitis, and infections of the skin and soft tissues, for which it has traditionally been widely used as a natural remedy. Favourable reports have been found showing the efficacy of its topical use for nappy rash, atopic eczema, nappy dermatitis, and umbilical cord separation. In the mouse model, it has been found to be effective in preserving corneal epithelial thickness in dry eye [74].

### 4.5. Human Faeces

In Traditional Chinese Medicine (TCM), the medical use of faecal matter (fresh faecal suspension or dry faeces) can be dated back to the fourth century, approximately 1700 years ago. Chinese doctors have accumulated unique and invaluable medical experience in the use of more than 50 different faecal materials [75,76]. TCM is integrated in today’s oriental society together with biomedicine as part of the culture and traditions of its civilisation. For this reason, the pharmacological potential of faeces has been studied on the basis of bibliographic reviews of TCM treatises, with the aim of determining its scientific and medicinal value [75,76]. Most of these faecal drugs have been insufficiently studied; however, fresh faecal juice (made from human faeces and known as “yellow soup”) is commonly used to treat food poisoning, severe diarrhoea, and unconsciousness due to high fever [76]. Zhang and colleagues [77] believe that the efficacy of this “soup” is mainly caused by the gut microbiota from fresh faecal water, and its principle for treating diseases is similar to the faecal microbiota transplantation method of modern medicine.

## 5. Other Ethnoveterinary Practices (Ritual Healing)

It is important to point out that like ethnomedicine, EVM includes a series of foundations based on experience, which can also incorporate rational foundations with elements of speculative scientific practices; in addition, practices exist that are based on magical–religious beliefs. Thus, we have recorded that in Galicia they used to treat scrofula in horses by washing them with the water in which a newlywed couple had previously washed themselves [36,41]. In Asturias, to facilitate fertility in cows, they would rub the animal with children’s urine [78].

Callejo and Iniesta [79] cite the existence of a boy in Yeste (Albacete) in the early 20th century who not only became famous for treating people, but also for treating horses affected by equine colic by simply rubbing the animal’s belly with his espadrille. This same practice was documented more recently by Ortega Madrid [80] in Cartagena (Murcia). In Villarino de los Aires (Salamanca), equine colic was treated by rubbing the belly of the sick animal with the hand of a person who had been born standing upright [81]; while in Guijo de Granadilla (Cáceres), it was believed that this condition was cured when the equine was ridden by a twin, and after having gone for a long ride [82].

Similarly, in another part of Cáceres province (Malpartida de Plasencia), when a horse was suffering from urine retention, the ritual of lifting a twin child on to its rump was performed. In a short time, the animal was fine [49].

However, the greatest example in Spain of the need to resort to persons who met certain birth requirements (e.g., being the seventh son of seven brothers, the older of two twin brothers) can be found in the performances of traditional healers known as *saludadores*. These healers, endowed with supposed power, were dedicated to healing persons and animals affected by rabies with their saliva, their breath, and certain prayers [80,83,84,85,86].

These *saludadores*, in order to be considered as healers and qualified to perform their duties, had to meet the aforementioned birth requirements, as well as passing various tests. Some of these tests involved walking on a red-hot iron in bare feet or extinguishing a burning ember with their tongue without making any mark on the performer [85].

Well-documented since the 15th century, the activity of the *saludadores* was performed throughout almost the whole of Spain until the middle of the 20th century, enjoying the approval of society as a whole, including the authorities [84,85,86]. There are numerous references in the Spanish press of the 20th century concerning the work of *saludadores* in treating people [86], but few references to their attempts to treat domestic animals bitten by a rabid dog recorded for contemporary Spanish EVM. A singular event was documented in 1907 in Valdanzo (Soria), where a case of rabies occurred in a donkey. The *Junta de Sanidad* (Health Board) of that locality adopted as a public health measure the cremation of the affected donkey and the isolation of the other domestic animals owned by the donkey’s owner, but this man ignored the agreements, having instead put himself into the hands of an itinerant *saludador* who came to the village [85]. To treat sheep bitten by a rabid dog in Talayuela and Navalmoral de la Mata (Cáceres), the work of the *saludador*, male or female, was reduced to giving the sick animal pieces of bread mixed with saliva, to the accompaniment of a ceremony with plenty of prayers and incantations [37].

On some occasions, women played an important and special role in magical–religious practices. For example, when tending to *formigo* in horses (hoof rot), it was treated with hot quartz, and if this prescription failed, the blessings of women were resorted to. The procedure consisted of placing the hoof on a clod of dirt and cutting the shape around it with a knife as the corresponding spell was incanted or warm blessings were pronounced invoking “the power of God and the Virgin Mary”. Then the clod was placed in the sun and, as it dried, the animal was cured of its illness [51]. Although, in that case, no human product was involved, there are occasions when the gender nuance is linked to various human products such as sweat or menstrual blood. As an example, we can cite the curing in Galicia of colic in cows, in which the sore area is rubbed with a woman’s used shirt. Afterwards, the garment has to be washed by another woman who is menstruating and the sick animal is given the water from the washing to drink [51]. To conclude these gender-related practices, we can mention that in Extremadura, it has been a widespread belief that snakes in general, and vipers in particular, escape the unpleasant odour given off when women’s hair is burnt. That is why, to prevent bites from vipers, many shepherds resorted to making such bonfires [37].

## 6. Historical Considerations, Ethnomedical and Cross-Cultural Comparison

The use of faeces and urine for therapeutic purposes has a long tradition in the history of veterinary medicine. However, we must start from a Dreckapotheke or copropharmacy, employing unclean materials, which was used in the same way in both medicine and veterinary medicine [87,88]. There is no doubt that these therapeutic products were widely used throughout history and across all cultures [89,90,91,92,93,94].

A synthetic and summarised view of the therapeutic uses of these works can put into historical perspective some medicines that a priori are difficult to understand, but which had a very elaborate construction through both magical–religious and empirical–rational elements that in their context provide therapeutic functionality. At least five works could be fundamental in capturing the essence and basics of these products: (i) the *Naturalis Historia* of Pliny the Elder; (ii) the *Alphita* glossary produced at the School of Salerno; (iii) *The Twelve books of Agriculture* of Lucius Junius Moderatus Columella; and (iv) The work of Christian Franz Paullini, the first edition of which dates from 1696, the title of which translated into English (“The dirt pharmacy”) is worth noting:

“Heylsame Dreck-Apotheke, wie nemlich mit Koth und Urin fast alle, ja auch die schwerste, gifftigste Kranckheiten und bezauberte Schaden, vom Haupt bis zu den Füssen, inn- und eusserlich, glücklich curirt worden, durch und durch nit allerhand curieusen, so nützlich- als ergetzlichen Historien und Anmerkungen, auch andern feinen Denckwürdigkeiten, bewährt und erläutert”.

[Holy Dirt Pharmacy–Holy filthy pharmacy, like almost all of them with faeces and urine, yes even the most severe, most toxic diseases and bewitching injuries, from head to toe, inside and out, happily curled, thoroughly curious with all sorts of things, so useful—Proven and explained as delightful histories and comments, also other fine memorable items].

Moving on to comment on some therapeutic applications, we can note that the Roman author Pliny the Elder, in volume V, book 28, of his encyclopedic work *Naturalis Historia*, has a section called *Ex homine remedia* dedicated specifically to medicines of human origin. It should be mentioned that he rejects them on anthropocentric grounds, as this would turn man into an animal [95]. However, if we focus on the use of urine in the Graeco–Roman world, we can observe that it was of great importance in therapeutics, as it was considered to be a product with great medicinal and magical qualities. In such a way that it was prescribed both as a simple medicine and as a compound, forming a part of pharmacological ingredients with empirical and belief-based implications, or in a rational way with scientific foundations, but also incorporating magic [95,96,97]. In general, it is not possible to determine the specific use of urine in veterinary practice which differentiates it from medicine. However, a review of the main authors shows its dermatological use throughout history [58,98,99], including in contemporary Spanish ethnomedicine in which the use of urine is still current [100]. However, Lucius Junius Moderatus Columella prescribed warm ox urine to treat abscesses once the pus had been extracted, and in some cases after cauterisation, he recommended washing with human urine that should be stale (Columella, *RR*. 6, 11, l). The remedies were used in the 19th century on the recommendation of Álvarez de Sotomayor Rubio, who translated *The Twelve Books of Agriculture*, written in Latin by Columella [101]. This Roman soldier and farmer advocated the use of radical remedies to treat scabies in horses, which he then washed with urine; he also used men’s rancid urine burnt on hot tiles on sheep after applying *alpechín*—the liquid from soaking olives—boiled until two thirds of it was absorbed. It is curious that he recommended the use of ox urine, not human urine, in stomach diseases, to heal cauterised wounds, and in curative practices for diarrhoea or dysentery [98]. On the other hand, Pliny the Elder did use urine from oxen and human urine for diarrhoea of bees consuming nectar from plants of the genus *Euphorbia* [95,98]. In cases of pneumonia in sheep, Columella, following Celsus, administered a recipe consisting of “instilling with a small horn [...] rancid and reheated human urine through the left nostril accompanied by a sixth of a portion of pork lard that was introduced down the throat” [98,102]. He also used rancid human urine to treat jaundice in livestock, and warmed and applied it to the beaks of chickens contracting the pip [98,101].

Another outstanding product of the Dreckapotheke was blood, whose use is widespread in therapeutics since the earliest times of humanity. However, menstrual blood has had very particular therapeutic connotations, especially because, since time immemorial, menstruation has had negative connotations that inspired fear and dread. Various authors suggest that in prehistoric times, menstrual blood was associated with contamination and attacks from hungry animals on hunters. On the other hand, the cycles of the moon had a mysterious correspondence with women’s menstrual cycles, as well as the influence of the moon on the tides. Hence, it could be justified that in humoral medicine, the same influence was exerted on the liquids and humours of the body. Menstruation could therefore be evidence of the movements of fluids due to the moon [103,104] and could thus be used in the balancing of humours. In the Graeco–Roman world, however, it was considered that menstrual blood contained harmful and damaging residues. Thus, in the 1st century, the magical beliefs that associated it with a dangerous element for people, plants, and animals were well established [104]. Hence, if we take into account the Hippocratic aphorism *similia similibus curantur* (“like is cured by like”), it is easy to understand that menstrual blood could be seen as a resource to treat poisoning or cultural diseases such as the “evil eye” following that philosophy of nature by which the very thing that caused the harm would cure it. The evident relationship of Greek medicine and veterinary medicine to magic can be seen in the therapeutic resources such as menstrual blood and the other human products [27,96]. However, there are also empirical belief positions, together with rational approaches, in the arguments of some Graeco–Roman authors. Pliny the Elder states that menstrual blood, dried and reduced to powder by adding soot and wax, cures the ulcers of all beasts of burden [95]. In ancient Rome, women’s menstrual blood was claimed to be excellent against haemorrhages [105]. This is reminiscent of the use of opotherapeutic drugs in the 19th and early 20th century, in which, for example, blood powder was used orally [105,106,107].

There is a wealth of data from the Middle Ages recorded for all these products [108]. It is worth noting that the School of Salerno produced the most important inventory of drugs in the early Middle Ages, which includes the use of medicines based on parts of the human body [109,110]. All of these remedies were passed down in the succeeding centuries. Already in the Modern Age [111], and more specifically in the second half of the last century [25], there were data on the widespread use of menstrual blood to cure warts in Spain. In contemporary Spanish ethnomedicine, it is still a relict and marginal practice [47,112,113,114,115,116]. It was also believed to be effective in attacks of hysteria [25] and was used to treat styes, with some local particularities in Extremadura: in Garrovillas, they state that it must be from a mother of twins; in Zalamea de la Serena, they say that it must be from the first period that a woman has after giving birth; and in Trujillo, they state that it must belong, provided that it is a woman, to the same menstruating woman [114].

In contemporary Spanish ethnomedicine, it is used to treat infections and to heal wounds. In fact, some informants have currently reported that they have used it with positive results in ear infections caused by earrings. However, there are regional nuances, and so in Asturias it is necessary to secrete saliva on waking [117], but in Extremadura it is indifferent [116]. It has also been used for trauma accompanied by ritual phrases [116], and to eradicate warts [25,116,117].

Women’s milk as a remedy is well documented in Graeco-Latin culture and Pliny the Elder, in his *Naturalis Historia*, describes the mixture of women’s milk with tortoise meat that was administered drop by drop for earache. In reality, it is a widespread remedy that could be found in any European country, given the great influence of Greek and Roman medicine. In Spain, it was widespread in the popular medicine of the province of León [118] and the Basque Country [119], where there are many references to this remedy, especially for very intense pain, saying that it quenched the thirst of the worm that was inside the ear. The use of women’s milk for acute otitis was common in Extremadura, where González Pozuelo [120] quotes it in his work “Cultural features of Extremadurean society”. He indicates that women’s milk was collected in a thimble and that if the painful ear was a girl’s, the woman’s milk should be from a woman raising a boy child and the other way round if it was a boy’s ear. Although other authors did not record these conditions, except that it had to be applied in the morning on an empty stomach and only for one week; however, the usual practice is that the time is indeterminate and it is used when it hurts and for as long as the complaint lasts. The naturalistic idea that otitis has an etiological agent, that it is a worm that penetrates through orifices and ends by destroying them, occupies an important place in European folk medicine. According to Castillo de Lucas [25], in the Basque Country and in Galicia, as in the case of Extremadura, it has to be a woman raising a child of the opposite sex to that of the sick person. In Asturias, it had to be milk that was suckled by a male [117]. Informants of recent studies report that this remedy makes them better and so they pass it on through conversations in a clear cultural diffusion throughout the region, as they are people who live in a village created by recent colonisation [116].

Ancient peoples used excrement regularly, including in particular the faeces of pregnant women, for all sorts of diseases [105]. As mentioned in the previous section, TCM has used an important arsenal of faecal medicines. In the case of human excrement, regardless of gender, it has been used as an emetic [75], and the previously mentioned “yellow soup” currently used as already indicated to treat food poisoning, severe diarrhoea, and unconsciousness due to high fever [76].

The work of Christian Franz Paullini (1696) is a unique book in that it provides data on how to use human and animal excrement to treat internal or external diseases; we could call it a faecal bacteriotherapy [121]. In fact, some studies show that faecal microbiota therapy is better than vancomycin for the treatment of recurrent *Clostridium* difficile infection, although the results of their study should be interpreted with caution [122]. These ancestral traditions have also reached contemporary Spanish ethnomedicine, where human excrement has been used to treat wounds, warts, malaria, toothache, and spider bites [25,47,123,124].

Regarding EVM in other countries, we found two references to the veterinary use of human urine in Italy. In central Lucania, urine was topically applied together with flowers of weaver’s broom (*Spartium junceum* L.) and the aerial parts of groundsel (*Senecio vulgaris* L.) against snake bites in cattle, equines, and sheep [125]. In Monte Acuto (Sardinia), urine was used on sheep as a disinfectant for pimples [126]. There are only a few other references to its use in Europe. In the Ukrainian side of Bukovina, urine has been applied to clean and treat large wounds in cattle [127], and in Liubań district (Belarus), to treat mastitis in cows and to disinfect wounds in horses [128]. In contrast, in the state of Paraíba (Brazil), internal remedies have been recorded. Here, cattle poisoned by feeding on cassava (*Manihot esculenta* Crantz) are forced to drink human urine. The preparation is quite simple, requiring the dilution of a cup of urine in 3 litres of water and mixing well [19]. In addition, as has been documented by Souto and colleagues [129], urine has been used curiously “to tame angry animals” (cattle, equines, goats, or sheep). Further, in this state in NE Brazil, Souto and colleagues [20] recorded the topical use (direct application) of human breast milk or mothers’ milk to heal eye inflammations in cattle. Finally, in relation to the veterinary use of human excrement, Landau and colleagues [130] documented that the Arad shepherds of the Golan Heights (Israel) treated the infectious ocular lesion known as white eye (corneal opacity) by dusting the ocular globes of their goats with powdered human faeces (using a straw). For their part, the Tuareg nomadic herders of the Agadez Region (Niger) traditionally treated different contagious skin necroses (e.g., corynebacteriosis, staphylococcosis) in their dromedaries using the application of human excrement to the wound [131]. In Tabasco State (Mexico), in the event of a domestic animal being bitten by a bat, the recommended remedy was to place a children’s nappy with baby faeces on it over the area of the bite [132].

## 7. Conclusions

The use of human products in EVM reveals the parallels between animals and animal ethnomedicine through a process of biocultural transmission throughout history. In this regard, we wish to emphasise what can be called the “Ethnobiology of Health” [133], an approach to the relationships between human beings, animal health, and environmental health that transcends the “One Health” concept. As such, in addition to conserving, compiling, and preventing the erosion of the rich ethnobiological heritage available, there is an urgent need to train researchers who cannot only understand the biological or ecological elements but also elucidate the social, cultural, and historical factors involved in these relationships. In this sense, the veterinary uses that we have just described and analysed must be contemplated from the system of thought from which they were constructed. This view is fundamental to the development of therapeutic tools and creating teaching and learning processes in new ethnoveterinary practices adapted to the users and those who demand them, at the same time preventing this heritage from disappearing. We believe that the way to achieve this from the perspective of researchers is transdisciplinary training, since interdisciplinarity as a model for innovation and the resolution of ethnoveterinary problems, and ethnobiological problems in general, produces areas that cannot be explained by the combination of specialities [134]. Our work is yet another example of how traditional knowledge in Spain has many aspects based on the idiosyncrasies and worldview of its people. Thus, from an anthropological point of view, EVM is framed in an ideological–symbolic subsystem of socio-cultural relations and enculturation processes throughout history.

Undoubtedly, the veterinary use of human urine is a very interesting example, in accordance with the high number of usage data recorded for the Spanish ethnomedicine [100]. However, we are struck by the large number of other types of remedies known in the field of ethnomedicine as opposed to those used in veterinary medicine, a fact also found in the EVM of other countries and regions, as we have shown in a cross-cultural comparison. Therefore, and taking into account the clear parallels between ethnoveterinary and ethnomedicine, we consider that it would be highly appropriate to carry out EVM fieldwork using ethnomedical data as an anamnesis of remedies and to rediscover them in veterinary applications. We believe that it is important to reveal that in our databases we have unpublished records, except for urinotherapy, of the medical use of a large number of products and human body parts, collected from perspectives ranging from magical beliefs to empiricism. Therefore, we have recorded data on cerumen, umbilical cord, the skull, fingers, teeth, sputum, vaginal discharge, hand fat, bones, hands, eyes, the navel, ears, hair, the placenta, and nails, in addition to those mentioned in this paper and currently comprising more than 220 records throughout Spain. Certainly, the use of human medicines, the ancestral origin of which is linked to magic and the astral use of body parts, continued in Greece, Rome, the Middle Ages, and the Modern Age, remaining in popular lore to this day in many cases. In view of this, it is worth noting that these drugs were included in modern works such as the *Dictionnaire universel des drogues simples* of Nicolas Lémery (1760). Therefore, we cannot disregard the symbolic value of these medicines, but must dwell on them, even if we know that there are diachronic aspects to their use from a rational or scientific perspective, which should not be considered simplistically as if they were a “pharmacy of horror”.

For all of the above, local veterinary knowledge must be intensively explored. We must assume that it is comparable to ethnomedical knowledge in terms of the number of human parts used and their therapeutic, symbolic and magical use. As our work progresses, we hope that new epistemological approaches from the ethnoveterinary and ethnomedical fields will emerge from theoretical debates on health.

Finally, we cannot end without remembering that where individuals do not have ready access to medicine —particularly in developing countries—knowledge, skills, and practices based on the theories and experiences indigenous to different cultures, whether explicable or not, are used in the maintenance of health as well as in the prevention, improvement, and treatment of illness. As such, even in this country, the lack of sanitary means for centuries meant that peasants had to resort to nature to make up for this deficit through popular knowledge. Their experience in bringing together products of all sorts, including human products, should be considered and respected as cultural heritage, as well as for the construction of new ways of understanding a holistic veterinary medicine in its scientific, social, historical, and anthropological aspects.

## Figures and Tables

**Table 1:** Human products used in contemporary Spanish ethnoveterinary medicine.

Product Used	Diseases or Troubles Treated	Animal (s) Treated	Administration Route ^1^	Geographical Location	References
Urine	Anthrax	Sheep, goats	IN	Valsequillo (Gran Canary Island)	Siemens Hernández [33]
	Cough	Piglets	IN	Basque Country	Thalamas [34]; Goicoetxea [35]
		Cows	IN	Galicia	Vázquez Varela [36]
	Mastitis	Ewes	EX	San Mateo (Gran Canary Island) and Extremadura	Siemens Hernández [33]; Domínguez Moreno [37]
	Wounds and cracks in the udders	Ewes	EX	Extremadura	Domínguez Moreno [37,38]
	Lice	Sheep	EX	Extremadura	Domínguez Moreno [37,38]
	Cutaneous myiasis	Cows	EX	Valle de Carranza (Vizcaya)	Peña [39]
	Mange	Sheep, goats	EX	Anguiano (La Rioja)	Elías and Muntión [40]
	Pinkeye	Cows	EX	Galicia	Vázquez Varela [36]; Lis Quibén [41]
	Eye injuries	Sheep	EX	Northern part of the province of Cáceres	Domínguez Moreno [37,38]
	Wounds and chafing	Cattle, equines, sheep	EX	A Pastoriza (Lugo), Bardenas Reales (Navarra), Comarca de La Campiña (Guadalajara), Aldearrubia (Salamanca), and Extremadura	Domínguez Moreno [37,38]; Seoane Veiga [42]; Blanco [43]; Hualde Pascual and Ormazábal Herraiz [44]; Orduna Portús and Mateo Pérez [45]
	Ulcers	Sheep	EX	Extremadura	Domínguez Moreno [37]
	Burns	Sheep	EX	Extremadura	Domínguez Moreno [37,38]
	Poisoning	All livestock	IN	Extremadura	Penco [46]
Menstrual fluid	Equine colic	Equines	IN	Comarca de la Sierra de Cádiz (Cádiz), Malpartida de Plasencia (Cáceres), and Asturias and Galicia	Vázquez Varela [36]; Lis Quibén [41]; Álvarez Peña [47]; Mata Moreno et al. [48]; Tejerina Gallardo [49]; Risco [50]; Freire Sánchez [51]
	Wounds and chafing	Equines	EX	Comarca de Monzón (Huesca)	Ferrández and Sanz [52]
	Lumps on ears	Equines	EX	Comarca de Monzón (Huesca)	Ferrández and Sanz [52]
	Warts	Cows	EX	Galicia	Vázquez Varela [36]
Saliva	Scald, hoof rot	Sheep	EX	Enclave de Treviño (Burgos)	Aguirre Sorondo et al. [53]
	Mange (sheep scab)	Sheep	EX	Zarza de Granadilla (Cáceres)	Domínguez Moreno [37,38]
	Corneal ulcers	Sheep, goats	EX	Tierra de Cameros (La Rioja), Campo de Montiel (Albacete), and Cueva Corcho (Gran Canary Island)	Siemens Hernández [33]; Elías and Muntión [40]; Morcillo et al. [54]
Breast milk	Otalgia	Sheep	EX	Extremadura	Domínguez Moreno [37]
Excrement	Wounds and chafing	Equines	EX	Serradilla (Cáceres)	Tejerina Gallardo [49]
	Meteorism	Cows	IN	Cantabria	García-Lomas [55]

^1^ Administration route: IN = internal use; EX = external use.

## Data Availability

All data are included as part of the manuscript.

## References

[1] Mathias E. (2004). Ethnoveterinary medicine: Harnessing its potential. Vet. Bull..

[2] Wanzala W., Zessin K.H., Kyule N.M., Baumann M.P.O., Mathias E., Hassanali A. (2005). Ethnoveterinary medicine: A critical review of its evolution, perception, understanding and the way forward. LRRD.

[3] Abo-EL-Sooud K. (2018). Ethnoveterinary perspectives and promising future. Int. J. Vet. Sci. Med..

[4] Abdalla M.A., McGaw L.J., McGaw L.J., Abdalla M.A. (2020). Introduction. Ethnoveterinary Medicine: Present and Future Concepts.

[5] Usha S., Rajasekaran C., Siva R. (2016). Ethnoveterinary medicine of the Shervaroy Hills of Eastern Ghats, India as alternative medicine for animals. J. Tradit. Complement. Med..

[6] Aziz M.A., Khan A.H., Pieroni A. (2020). Ethnoveterinary plants of Pakistan: A review. J. Ethnobiol. Ethnomed..

[7] Sikarwar R.L.S., Tiwari A.P. (2020). A review of plants used in ethnoveterinary medicine in Central India. Indian J. Tradit. Knowl..

[8] McGaw L.J., Famuyide I.M., Khunoana E.T., Aremu A.O. (2020). Ethnoveterinary botanical medicine in South Africa: A review of research from the last decade (2009 to 2019). J. Ethnopharmacol..

[9] Eiki N., Sebola N.A., Sakong B.M., Mabelebele M. (2021). Review on Ethnoveterinary practices in Sub-Saharan Africa. Vet. Sci..

[10] Borges A.K.M., Barboza R.R.D., Souto W.M.S., Alves R.R.N., McGaw L.J., Abdalla M.A. (2020). Natural remedies for animal health in Latin America. Ethnoveterinary Medicine: Present and Future Concepts.

[11] Mayer M., Zbinden M., Vogl C.R., Ivemeyer S., Meier B., Amorena M., Maeschli A., Hamburger M., Walkenhorst M. (2017). Swiss ethnoveterinary knowledge on medicinal plants–a within-country comparison of Italian speaking regions with north-western German speaking regions. J. Ethnobiol. Ethnomed..

[12] Bullitta S., Re G.A., Manunta M.D.I., Piluzza G. (2018). Traditional knowledge about plant, animal, and mineral-based remedies to treat cattle, pigs, horses, and other domestic animals in the Mediterranean island of Sardinia. J. Ethnobiol. Ethnomed..

[13] Marković M.S., Pljevljakušić D.S., Nikolić B.M., Miladinović D.L., Djokić M.M., Rakonjac L.B., Stankov Jovanović V.P. (2021). Ethnoveterinary knowledge in Pirot County (Serbia). S. Afr. J. Bot..

[14] González J.A., Verde A., Pardo-de-Santayana M., McGaw L.J., Abdalla M.A. (2020). The use of plants for animal health care in the Spanish Inventory of Traditional Knowledge. Ethnoveterinary Medicine: Present and Future Concepts.

[15] Volpato G., Lamin Saleh S.M., Di Nardo A. (2015). Ethnoveterinary of Sahrawi pastoralists of Western Sahara: Camel diseases and remedies. J. Ethnobiol. Ethnomed..

[16] González J.A., Amich F., Postigo-Mota S., Vallejo J.R. (2016). Therapeutic and prophylactic uses of invertebrates in contemporary Spanish ethnoveterinary medicine. J. Ethnobiol. Ethnomed..

[17] González J.A., Amich F., Postigo-Mota S., Vallejo J.R. (2016). The use of wild vertebrates in contemporary Spanish ethnoveterinary medicine. J. Ethnopharmacol..

[18] González J.A., Vallejo J.R. (2021). The use of domestic animals and their derivative products in contemporary Spanish ethnoveterinary medicine. J. Ethnopharmacol..

[19] Barboza R.R.D., Souto W.M.S., Mourão J.S. (2007). The use of zootherapeutics in folk veterinary medicine in the district of Cubati, Paraíba State, Brazil. J. Ethnobiol. Ethnomed..

[20] Souto W.M.S., Barboza R.R.D., Mourão J.S., Alves R.R.N. (2012). Traditional knowledge of *sertanejos* about Zootherapeutic practices used in ethnoveterinary medicine of NE Brazil. Indian J. Tradit. Know..

[21] Moog F.P., Karenberg A. (2003). Between horror and hope: gladiator’s blood as a cure for epileptics in ancient medicine. J. Hist. Neurosci..

[22] Nutton V. (2012). Ancient Medicine.

[23] Berry J.W., Poortinga Y.H., Segall M.H., Dasen P.R. (2002). Cross-Cultural Psychology: Research and Applications.

[24] Kottak C.P. (2020). Mirror for Humanity: A Concise Introduction to Cultural Anthropology.

[25] Castillo de Lucas A. (1958). Folkmedicina.

[26] Walker S.S. (1979). Witchcraft and healing in an African Christian church. J. Relig. Afr..

[27] Lloyd G.E.R. (1979). Magic, Reason, and Experience: Studies in the Origins and Development of Greek Science.

[28] Appadurai A. (1986). Theory in Anthropology: Center and Periphery. Comp. Stud. Soc. Hist..

[29] Glik D.C. (1988). Symbolic, ritual and social dynamics of spiritual healing. Soc. Sci. Med..

[30] Csordas T.J., Lewton E. (1998). Practice, performance, and experience in ritual healing. Transcult. Psychiatry.

[31] Kahn L.H., Kaplan B., Monath T.P., Steele J.H. (2008). Teaching “one medicine, one health”. Am. J. Med..

[32] Gyles C. (2016). One Medicine, One Health, One World. Can. Vet. J..

[33] Siemens Hernández L. (1981). Veterinaria tradicional de cabras y ovejas entre los pastores de Gran Canaria. Anu. Estud. Atlánticos.

[34] Thalamas J. (1931). Contribución al estudio etnográfico del País Vasco continental. Anu. Soc. Eusko-Folk..

[35] Goicoetxea A. (2011). Veterinaria Popular en el País Vasco.

[36] Vázquez Varela J.M. (2003). Introducción á Antropoloxía da Veterinaria Popular en Galicia.

[37] Domínguez Moreno J.M. (1994). La etnoveterinaria en Extremadura: El tratamiento del ganado lanar. Rev. Folk..

[38] Domínguez Moreno J.M., Rodríguez Becerra S., Coordinador (1993). Aspectos populares de la profilaxis y la curación del ganado ovino en Extremadura. Actas del Simposio “Trashumancia y Cultura Pastoril en Extremadura”.

[39] Peña L.M. (1991). Veterinaria y conocimientos populares sobre el ganado vacuno en el Valle de Carranza. Anu. Eusko-Folk..

[40] Elías L.V., Muntión C. (1989). Los Pastores de Cameros.

[41] Lis Quibén V. (1949). La medicina Popular en Galicia.

[42] Seoane Veiga Y. (2002). Primeras aproximaciones a un estudio de veterinaria popular en A Pastoriza (Lugo). Boln. Mus. Prov. Lugo.

[43] Blanco J.F. (1985). Medicina y Veterinaria Populares en la Provincia de Salamanca.

[44] Hualde Pascual C., Ormazábal Herraiz A. (2002). Usos y prácticas de medicina y veterinaria popular en la Campiña de Guadalajara. Cuad. Etnol. Guadalaj..

[45] Orduna Portús P.M., Mateo Pérez M.R. (2018). Etnoveterinaria trashumante navarra. Un nexo de unión entre los pastores dela montaña y la Bardena Real. Rev. Cent. Estud. Merind. Tudela.

[46] Penco A.D. (2005). Medicina Popular Veterinaria en la Comarca de Zafra. Ph.D. Thesis.

[47] Álvarez Peña A. (2004). Melecina Máxico-Tradicional n’Asturies.

[48] Mata Moreno C., Maurer P., Rodríguez Estévez V., Fernández Reyes A. (2004). Recopilación del Conocimiento Ganadero Tradicional de la Comarca de la Sierra de Cádiz y su Validación Para la Reconversión e Implantación de la Ganadería Ecológica.

[49] Tejerina Gallardo A. (2010). Usos y Saberes Sobre las Plantas de Monfragüe. Etnobotánica de la Comarca Natural.

[50] Risco V. (1935). Folklore de Coaledro (Ourense). Nós.

[51] Freire Sánchez P. (2006). Menciñeiros, Saludadores e Compoñedores: Los Sanadores en la Medicina Popular de Galicia.

[52] Ferrández J.V., Sanz J.M. (1993). Las Plantas en la Medicina Popular de la Comarca de Monzón (Huesca).

[53] Aguirre Sorondo A., Sáenz de Urturi I., Garamendi L.S., González Salazar J.A., Galdos J.J. (2006). Etnografía del Enclave de Treviño, II.

[54] Morcillo T., López M., Fajardo J. (2020). Y Estaban el Pastor, el Perro y la Garrota… El Pastoreo Tradicional en el Campo de Montiel.

[55] García-Lomas G.A. (1993). Mitología y Supersticiones de Cantabria: Materiales y Tanteos Para su Estudio.

[56] Sarigul N., Korkmaz F., Kurultak I. (2019). A new artificial urine protocol to better imitate human urine. Sci. Rep..

[57] Bouatra S., Aziat F., Mandal R., Guo A.C., Wilson M.R., Knox C., Bjorndahl T.C., Krishnamurthy R., Saleem F., Liu P. (2013). The human urine metabolome. PLoS ONE.

[58] Marcos Casquero M.A. (2005). Virtudes mágicas y medicinales de la orina según los escritores latinos (1ª parte). Estud. Hum. Filol..

[59] Ramesh H.A., Azmathulla M., Baidya M., Asad M. (2010). Wound healing activity of human urine in rats. Res. J. Pharm. Biol. Chem. Sci..

[60] Fu Y., Guan J., Guo S., Guo F., Niu X., Liu Q., Zhang C., Nie H., Wang Y. (2014). Human urine-derived stem cells in combination with polycaprolactone/gelatin nanofibrous membranes enhance wound healing by promoting angiogenesis. J. Transl. Med..

[61] Uzieliene I., Urbonaite G., Tachtamisevaite Z., Mobasheri A., Bernotiene E. (2018). The potential of menstrual blood-derived mesenchymal stem cells for cartilage repair and regeneration: Novel aspects. Stem Cells Int..

[62] Cuenca J., Le-Gatt A., Castillo V., Belletti J., Díaz M., Kurte G.M., González P.L., Alcayaga-Miranda F., Schuh C.M.A.P., Ezquer F. (2018). The reparative abilities of menstrual stem cells modulate the wound matrix signals and improve cutaneous regeneration. Front. Physiol..

[63] Dalirfardouei R., Jamialahmadi K., Jafarian A.H., Mahdipour E. (2019). Promising effects of exosomes isolated from menstrual blood-derived mesenchymal stem cell on wound-healing process in diabetic mouse model. J. Tissue Eng. Regen. Med..

[64] Chen L., Qu J., Xiang C. (2019). The multi-functional roles of menstrual blood-derived stem cells in regenerative medicine. Stem Cell Res. Ther..

[65] Vila T., Rizk A.M., Sultan A.S., Jabra-Rizk M.A. (2019). The power of saliva: Antimicrobial and beyond. PLoS Pathog..

[66] Abiko Y., Nishimura M., Kaku T. (2003). Defensins in saliva and the salivary glands. Med. Electron Microsc..

[67] Khurshid Z., Naseem M., Asiri F.Y.I., Mali M., Khan R.S., Sahibzada H.A., Zafar M.S., Moin S.F., Khan E. (2017). Significance and diagnostic role of antimicrobial cathelicidins (LL-37) peptides in oral health. Biomolecules.

[68] Oudhoff M.J., Bolscher J.G.M., Nazmi K., Kalay H., Hof W., Amerongen A.V.N., Veerman E.C.I. (2008). Histatins are the major wound-closure stimulating factors in human saliva as identified in a cell culture assay. FASEB J..

[69] Torres P., Castro M., Reyes M., Torres V.A. (2018). Histatins, wound healing and cell migration. Oral Dis..

[70] Wisner A., Dufour E., Messaoudi M., Nejdi A., Marcel A., Ungeheuer M.N., Rougeot C. (2006). Human opiorphin, a natural antinociceptive modulator of opioid-dependent pathways. Proc. Natl. Acad. Sci. USA.

[71] Rodrigues Neves C., Buskermolen J., Roffel S., Waaijman T., Thon M., Veerman E., Gibbs S. (2019). Human saliva stimulates skin and oral wound healing in vitro. J. Tissue Eng. Regen. Med..

[72] Wambach K., Riordan J. (2015). Breastfeeding and Human Lactation.

[73] Arnardottir H., Orr S.K., Dalli J., Serhan C.N. (2016). Human milk proresolving mediators stimulate resolution of acute inflammation. Mucosal Immunol..

[74] Witkowska-Zimny M., Kamińska-El-Hassan E., Wróbel E. (2019). Milk therapy: Unexpected uses for human breast milk. Nutrients.

[75] Frick J. (1957). Medicinal uses of substances derived from the animal organism (in Tsinghai). Anthropos.

[76] Du H., Kuang T.T., Qiu S., Xu T., Huan C.L.G., Fan G., Zhang Y. (2019). Fecal medicines used in traditional medical system of China: A systematic review of their names, original species, traditional uses, and modern investigations. Chin. Med..

[77] Zhang F., Cui B., He X., Nie Y., Wu K., Fan D., Feng B., Chen D., Ren J., Deng M. (2018). Microbiota transplantation: Concept, methodology and strategy for its modernization. Protein Cell..

[78] Fernández-Guisasola Muñiz F.J., Fernández García J. (2012). Remedios d’orixe humanu na melecina popular asturiana (usu real y simbólicu) / Remedies of human origin in Asturian folk medicine (real and symbolic use). Lletres Astur..

[79] Callejo J., Iniesta J.A. (2001). Testigos del Prodigio: Poderes Ocultos y Oficios Insólitos.

[80] Ortega Madrid J. (2015). El último saludador. Murgetana.

[81] Morán Bardón C. (1927). Creencias sobre curaciones supersticiosas recogidas en la provincia de Salamanca. AMSEAEP.

[82] Hurtado P. (1902). Supersticiones Extremeñas. Anotaciones Psico-Fisiológicas.

[83] Aguirre Sorondo A. (1990). Los saludadores. Cuad. Etnogr. Navar..

[84] Peris Barrio A. (2009). Los saludadores. Rev. Folk..

[85] Poza P. (2012). Los saludadores y su actividad en España. Inf. Vet..

[86] Poza P. (2013). La prensa histórica como testigo de la rabia y la actividad de los saludadores. Inf. Vet..

[87] Paullini C.F. (1734). Neu-Vermehrte Heylsame Dreck-Apothecke, wie Nemlich mit Koth und Urin Fast Alle, ja auch Die Schwerste, Gifftigste Kranckheiten, und Bezauberte Schäden vom Haupt bis zun Füssen, inn- und Äusserlich, Glücklich Curiret Worden.

[88] Goede B. (2006). Die “Dreckapotheke” der Ägypter: Das Erwachen der Heilkunst im Alten Ägypten. Antike Welt.

[89] Thompson C.R. (1924). Assyrian medical texts. Proc. R. Soc. Med..

[90] Cuenca-Estrella M., Barba R. (2004). La Medicina en el Antiguo Egipto.

[91] Martins e Silva J. (2010). A medicina na Mesopotâmia antiga (2ª parte). Acta Médica Port..

[92] Unschuld P.U. (1986). Medicine in China: A History of Pharmaceutics.

[93] De la Cruz M. (1991). Libellus de Medicinalibus Indorum Herbis: Manuscrito azteca de 1552, Según Traducción Latina de Juan Badiano.

[94] Seelentag M.B. (2019). “Empirica” aus Tirol-Die Volksmedizinischen Heilverfahren bei Dr. Johann Franc (1649–1725): Transkription, Übersetzung und Kommentierung. Ph.D. Thesis.

[95] Cantó J., Gómez Santamaría I., González Marín S., Tarriño E. (2007). Plinio: Historia Natural.

[96] Edelstein L. (1937). Greek Medicine in its relation to religion and magic. Bull. Inst. Hist. Med..

[97] Fernández García A. (2006). La orina en las recetas de los alquimistas griegos: ‘Papiro X de Leiden’ y ‘Papiro de Estocolmo’. Estud. Clásicos.

[98] Marcos Casquero M.A. (2006). Virtudes mágicas y medicinales de la orina según los escritores latinos (2ª parte). Estud. Hum. Filol..

[99] Savica V., Calò L.A., Santoro D., Monardo P., Mallamace A., Bellinghieri G. (2011). Urine therapy through the centuries. J. Nephrol..

[100] Vallejo J.R., Aparicio Mena A.J., González J.A. (2017). Human urine-based therapeutics in Spain from the early 20th century to the present: An historical literature overview and a present-day case study. Acta Med.-Hist. Adriat..

[101] Álvarez de Sotomayor Rubio J.M. (1824). Los Doce Libros de Agricultura que Escribió en Latín Lucio Junio Moderato Columela.

[102] Blánquez A. (1966). Aurelio Cornelio Celso. Los Ocho Libros de la Medicina.

[103] Boorstin D.J. (1986). Los Descubridores.

[104] Benavides Iglesias J. (2009). La menstruación: Un asunto sobre la Luna, venenos y flores. Rev. Med. Univers..

[105] Amaral A. (1921). La Opoterapia hipofisaria en Obstetricia. Conferencia dada en sesión general el 20 de junio de 1921 por el Dr. Moisés Amaral (Médico del Hospital San Borja. Sociedad Científica de Chile). Notas y Mem. año treinta y uno.

[106] Sousa F.J.D. (1899). Algumas Palavras Sobre a Opotherapia: Tratamento de Certas Doenças por Extractos D’orgãos Animais.

[107] Vidal M.M. (1901). Noções Gerais de Opotherapia.

[108] Fresquet Febrer J.L., López Piñero J.M. (2001). El uso de los animales y de productos de origen animal en el tratamiento de las enfermedades. Los Animales en la Ciencia y la vida Humana. Ilustraciones de un Milenio (siglos XI–XX).

[109] Stiehler-Alegría G. (2007). Hatte die zootherapie ägyptischer und babylonischer pharmakopoen einfluss auf die dreck-apotheke des 17. Jahrhunderts?. ISIMU.

[110] Vallejo J.R., Cobos J.M. (2013). El recetario de la Escuela de Salerno conocido como el “Antidotarium Nicolai”. Med. Natur..

[111] Guichot Sierra A., Machado Álvarez A. (1883). Supersticiones populares recogidas en Andalucía y comparadas con las portuguesas. Biblioteca de las Tradiciones Populares Españolas.

[112] Guío Cerezo Y. (1992). Naturaleza y Salud en Extremadura: Los remedios.

[113] Fernández D., Breaux J. (1998). Medicina Popular, Magia y Religión en El Bierzo.

[114] Domínguez Moreno J.M. (2004). Dermatología popular en Extremadura (II). Rev. Folk..

[115] Pardo de Santayana M. (2008). Estudios Etnobotánicos en Campoo (Cantabria). Conocimiento y Uso Tradicional de Plantas.

[116] Vallejo J.R., Peral D., Carrasco M.C. (2008). Prácticas mágicas en la medicina popular de un pueblo extremeño de colonización. Gaz. Antropol..

[117] Junceda Avello E. (1987). Medicina Popular en Asturias.

[118] Rúa Aller F.J., Rubio Gago M.E. (1990). La Medicina Popular en León.

[119] Goicoetxea A. (1983). Capítulos de la Medicina Popular Vasca.

[120] González Pozuelo F. (1985). Rasgos culturales de la sociedad tradicional extremeña. Cuad. Real. Soc..

[121] van Nood E., Vrieze A., Nieuwdorp M., Fuentes S., Zoetendal E.G., de Vos W.M., Visser C.E., Kuijper E.J., Bartelsman J.F., Tijssen J.G. (2013). Duodenal infusion of donor feces for recurrent Clostridium difficile. N. Engl. J. Med..

[122] van Schooneveld T.C., Gross A., Kalil A.C. (2013). Duodenal infusion of feces for recurrent Clostridium difficile. N. Engl. J. Med..

[123] García Arambilet L.A. (1990). Medicina Popular en la Provincia de Soria: Descripción y Análisis de sus Prácticas. Degree Thesis.

[124] Pérez Vidal J. (2007). Contribución al Estudio de la Medicina Popular Canaria.

[125] Pieroni A., Howard P., Volpato G., Santoro R.F. (2004). Natural remedies and nutraceuticals used in ethnoveterinary practices in inland southern Italy. Vet. Res. Commun..

[126] Piluzza G., Virdis S., Serralutzu F., Bullitta S. (2015). Uses of plants, animal and mineral substances in Mediterranean ethno-veterinary practices for the care of small ruminants. J. Ethnopharmacol..

[127] Sõukand R., Pieroni A. (2016). The importance of a border: Medical, veterinary, and wild food ethnobotany of the Hutsuls living on the Romanian and Ukrainian sides of Bukovina. J. Ethnopharmacol..

[128] Sõukand R., Hrynevich Y., Prakofjewa J., Valodzina T., Vasilyeva I., Paciupa J., Shrubok A., Hlushko A., Knureva Y., Litvinava Y. (2017). Use of cultivated plants and non-plant remedies for human and animal home-medication in Liubań district, Belarus. J. Ethnobiol. Ethnomed..

[129] Souto W.M.S., Mourão J.S., Barboza R.R.D., Mendonça L.E.T., Lucena R.F.P., Confessor M.V.A., Vieira W.L.S., Montenegro P.F.G.P., López L.C.S., Alves R.R.N. (2011). Medicinal animals used in ethnoveterinary practices of the ‘Cariri Paraibano’, NE Brazil. J. Ethnobiol. Ethnomed..

[130] Landau S.Y., Muklada H., Abu-Rabia A., Kaadan S., Azaizeh H. (2014). Traditional Arab ethno-veterinary practices in small ruminant breeding in Israel. Small Rumin. Res..

[131] Antoine-Moussiaux N., Faye B., Vias G.F. (2007). Tuareg ethnoveterinary treatments of camel diseases in Agadez area (Niger). Trop. Anim. Health Prod..

[132] Nava-Hernández G., Aldasoro-Maya E.M., Perezgrovas R., Vera-Cortés G. (2018). Interacciones del ser humano con animales de traspatio: Un estudio desde la Etnoveterinaria en Tabasco, México. Nova Sci..

[133] Quinlan M.B., Quinlan R.J. (2016). Ethnobiology in One Health. Ethnobiol. Lett..

[134] Brand F., Schaller F., Völker H. (2004). Transdisziplinarität. Bestandsaufnahme und Perspektiven. Beiträge zur Thesis-Arbeitstagung im Oktober 2003 in Göttingen.

